# Supplementary material for: Causal interplay between lactose intolerance and gut microbiota: a combined bidirectional Mendelian randomization and in vivo validation study
Source: Front Nutr. 2026 Jun 1;13:1803337. doi: 10.3389/fnut.2026.1803337 (PMC13265576; doi:10.3389/fnut.2026.1803337)
Supplement: Supplementary file 1 [file Data_Sheet_1.zip › supplementary materials/Forward/forest plot/ebi-a-GCST90027772.finngen_R12_E4_LACTONAS.pdf]

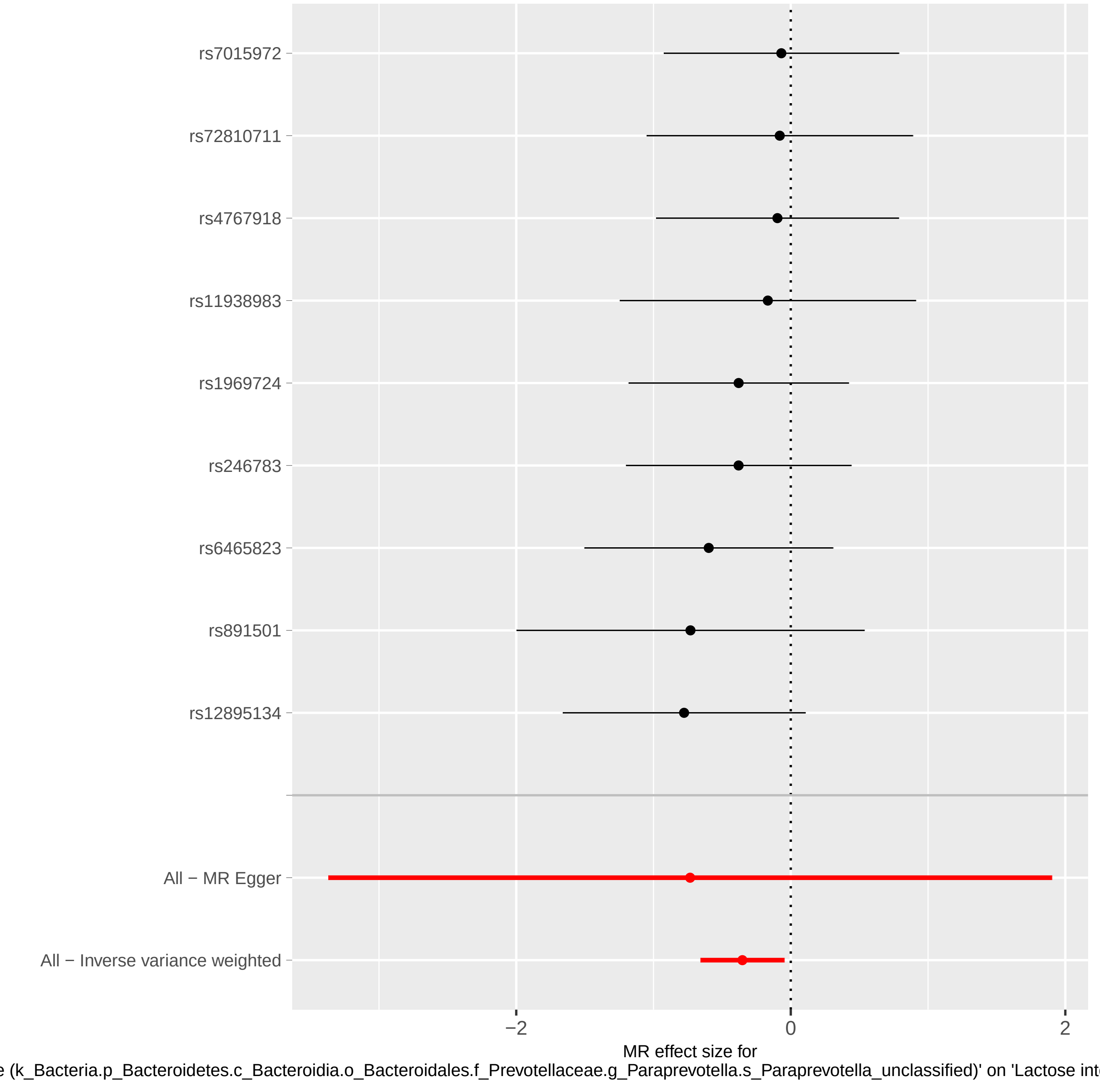
rs7015972
rs72810711
rs4767918
rs11938983
rs1969724
rs246783
rs6465823
rs891501
rs12895134
All − MR Egger
All − Inverse variance weighted
−2
0
2
MR effect size for
e (k_Bacteria.p_Bacteroidetes.c_Bacteroidia.o_Bacteroidales.f_Prevotellaceae.g_Paraprevotella.s_Paraprevotella_unclassified)' on 'Lactose int
